# Factors Affecting Parent’s Practice Regarding the Management of Children’s Fever

**DOI:** 10.7759/cureus.25658

**Published:** 2022-06-04

**Authors:** Badeah A Alsofyani, Noha S Hassanien

**Affiliations:** 1 Preventive Medicine, Saudi Board of Preventive Medicine, Taif, SAU; 2 Department of Biostatistics, High Institute of Public Health - Alexandria University, Alexandria, EGY

**Keywords:** saudi, doctor, parent worry, parental practice, knowledge, fever, children

## Abstract

Background

Fever is a common cause of doctor visits among children. Fever and its effects are a source of anxiety for parents. There is a discrepancy between parents’ understanding and actual practice, which is influenced by a range of variables. This study aimed to assess the factors that influence parents’ practices and home behaviors when their children had a fever.

Methodology

A cross-sectional study was conducted among 384 randomly selected Saudi parents attending primary healthcare centers of Taif city from February to April 2022. Data were collected using a pretested, self-designed questionnaire (α= 0.760) developed by the researcher through interviews. The questionnaire included questions on socio-demographic characteristics, five items to assess parents’ knowledge, and 12 items to assess parents’ evidence-based practices regarding childhood fever, level of worry and concerns, and difficulties encountered during management.

Results

About 43% of parents did not know the harmful degree of fever and 77% had a misbelief that fever is a harmful phenomenon. Non-evidence-based practices included the use of over-the-counter medications (in 76.6% of our sample) and unnecessary visits to health services (in more than 50%). Only 12% reported good practices. Half of the parents obtain their information about fever from doctors and 31.3% from the internet. The majority of parents reported high worry levels. Multiple linear regression analysis revealed that the most important significant predictors of parents’ practices are level of worry, source of information, knowledge, and mothers’ education level.

Conclusions

This study identified the gaps where parents’ practices regarding fever need education. Parents reported high levels of worry about the febrile child, which, in turn, led to poor practices such as unnecessary health visits and overuse of antipyretics. The study emphasized the importance of the role of doctors as the main source of parents’ information regarding fever. Hence, good informative communication between doctors and parents will decrease their worry and promote the practices of parents.

## Introduction

Fever is a common clinical complaint in children and one of the most recurrent presentations in primary healthcare clinics and emergency rooms. Although associated serious conditions with fever are less common, fever causes a burden as it leads to frequent emergency visits, hospital admissions, and laboratory investigations. Furthermore, it is a major concern for parents because they act as a major line of defense against anything that may harm their children or derail their development by threatening their physical, social, cognitive, and emotional health and safety. Fever is a common concern that caregivers must contend with as it is a frequent symptom of other infections and leads to repeated pediatric visits [1,2]. Parental management of childhood fever, febrile sickness, and other associated complications can be daunting, especially if one lacks the necessary knowledge [3,4]. Several studies have identified that the care of a feverish or febrile child may be motivated by fever phobia, which is an unrealistic fearful view of fever that causes parental discomfort, anxiety, apprehension, and distress [1,3,5]. Many studies have reported poor knowledge, perceptions, attitudes, and practices regarding childhood fever and its management [1,3,6].

High body temperature is usually identified subjectively by parents. It is noted from a child’s general appearance and is usually assessed by touching the child with the hand on the forehead, enabling parents to identify fever [1,3]. However, concerns by parents and doctors that the underlying illness may be different from initial conditions, in addition to red flags such as rapid breathing, cyanosis, and poor peripheral circulation, necessitating urgent care. Other features indicating severity include petechial rashes, diminished breath sounds, meningeal irritation, crackles on auscultation, decreased consciousness, and seizures. Still, several studies have affirmed that there is no need to fear, even though the lack of parental knowledge manages to elicit an overreaction leading to frequent hospital visits and other misconceptions and misperceptions about fever and its effects, such as misuse of two or more antipyretic drugs together and use of antibiotics to reduce high temperature[1,3]. Other consequences include calls to healthcare providers that may lead to unnecessary tests and prescriptions to alleviate caregiver anxiety, improper use of antipyretics and antibiotics, as well as physical fever measurement and management techniques [1,3]. Many studies have reported parental knowledge regarding fever in terms of its definition, its causes, methods of body temperature measurement, management practices, and treatment modalities [1,7,8]. Many of these investigations and others indicate that few parents can define fever while others have correct general information but lack specifics on temperature ranges [1,7,8]. The primary sources of parental knowledge include healthcare professionals, the internet, friends, and family, which raise the potential problem of mixed messages [2].

Research on complications of fever indicates that febrile convulsions or seizures are the most common and concerning for parents who believe and fear that untreated high fever may lead to other adverse consequences, such as loss of consciousness, coma, dehydration, brain damage, hearing loss, and death [1,4,5,7]. Though seizures are defined as benign common convulsive disorders accompanied by high body temperature without underlying severe health issues in children under age five, they invoke fear, anxiety, or even trauma [9]. The apprehension may explain poor fever management practices by many parents, whose focus on temperature reduction and monitoring, as well as administration of antipyretics is undermined by incomplete knowledge [1,7,10]. In the same vein, some studies also report problems of potential over and underdosing by parents, in addition to the use of relatively unsatisfactory traditional practices, for example, some parents use alcohol compressors to bring down a fever or use herbal remedies whatever the cause, which increase the risks associated with inappropriate drug use [2,11-13].

In summary, there are variations in parental knowledge, attitudes, perceptions, beliefs, and practices regarding childhood fever and its management. However, these elements are assessed as poor or deficient, which could undermine treatment efficacy [1,7]. Many scholars have identified the need for educational programs to empower caregivers in this area [3,6,11]. Clinicians and pharmacists can play a crucial instructive role [2]. Incidentally, a high level of education is correlated with lower levels of anxiety and reduced fever phobia [5]. This study aimed to measure parental knowledge and identify home practices regarding pediatric fever in Taif city, Saudi Arabia, in 2022.

## Materials and methods

Study design, setting, and sampling

A cross-sectional study was conducted among parents attending the primary healthcare centers (PHCs) of Taif city for reasons other than the sickness of their child. We estimated the minimum required sample size for the study to be 384 parents, assuming 50% good knowledge and practice regarding childhood fever among parents with 95% confidence limits and 5% precision using Epi Info 2002 software. A stratified sample technique was adopted in which the city was stratified into four geographic regions, with each region represented by one PHC. From each center, 96 parents were randomly selected using a systematic random sample of every third parent.

Data collection

Parents who agreed to participate were interviewed using a predesigned structured questionnaire developed from other previously similar validated and reliable surveys [1]. The questionnaire was reviewed by doctors and pediatricians for face validity. The questionnaire was tested in a pilot study of 30 parents revealing good reliability (Cronbach’s alpha = 0.760). The questionnaire consisted of four parts. Part one included personal and child characteristics (age of parents, education of parents, income of the family, occupation of the mother, number of the children, history of chronic disease or disability of the child, and history of febrile convulsions). Part two included five questions to assess the knowledge of the parents regarding fever (defining normal temperature, defining fever, defining high fever, defining the harmful degree of fever, and perceived adequacy of knowledge). Each response was coded as true and false. The correct answer was assigned 1 point while the wrong one was assigned 0 point. The five items were summed to obtain the total knowledge score. In addition, the participants were asked about their source of information. Part three included 12 questions to assess the practices of parents in home management of childhood fever and coded as 0 for inappropriate practice and 1 for appropriate practice based on guidelines of fever management [14] and the pediatrician’s opinion. All 12 items were summed to the total practice score. Participants were further classified into one of three groups based on their total practice score. The first group (poor practice) included parents below the first quartile of the practice score (below 8), the second group (fair practice) included parents with a score between the first quartile (score of 8) and the third quartile (score of 11), and the third group (good practice) included parents above the third quartile (above 11). Part four included questions about the concern of the harm of fever, level of worry, main medications used in reducing fever, and difficulties encountered by parents in administering the antipyretic.

Ethical issues

Institutional ethical approval was obtained from the King Abdelaziz City for Science and Technology, Institutional Review Board (IRB registration number: HAP-02-T-067; and approval number: 311). Participation was voluntary after obtaining approval from participants, and confidentiality was assured. The research was conducted in accordance with the Declaration of Helsinki.

Statistical analysis

Analyses were performed using SPSS version 22.0 (IBM Corp., Armonk, NY, USA). Categorical variables were summarized as frequencies and percentages. Descriptive statistics were calculated for quantitative variables as means and standard deviation (SD) or median and interquartile range (IQR). Data were tested for normality using the Shapiro-Wilk test. Non-parametric analysis was conducted; Mann-Whitney test (two groups difference), Kruskal-Wallis test (three or more groups difference), followed by the post-hoc test (Tukey) if there is a significant difference. Spearman correlation coefficient was used to measure the correlation between two quantitative variables. P-values of ≤0.05 was considered significant, and all tests had two-tailed hypotheses. All significant factors associated with total knowledge score and practices were entered in multiple linear regression analysis in which the dependent variables were total knowledge score and total practice score after testing the assumptions of regression analysis.

## Results

A total of 384 parents responded to the questionnaire, and their characteristics are shown in Table 1. The mean age of the mothers was 34 ± 7.9 years and of the fathers was 41 ± 9.1 years. About 78% of participants were females, and 82.8% of those who took care of children were mothers. More than half of the parents had a university or above level of education (65.4% for the mothers and 54.9% for the fathers). The percentage of the highest income (>9,000 thousand) was 48.2% and the lowest income (1,000-3,000) was 7.6%. The mean number of children for the study participants was 2.7 ± 1.7. About 6.0% of parents had a child with a disability or chronic disease, and 10.9% had a history of febrile convulsion.

**Table 1:** Characteristics of parents and children. SD: standard deviation

Characteristics	Frequency N = 384	Percent
Gender		
Male	84	21.9
Female	300	78.1
Baby care		
Mother	318	82.8
Father	60	15.6
Sister	6	1.6
Age of mother in years (mean ± SD)	34 ± 7.9	
Education of mother		
Below university	133	34.6
University and above	251	65.4
Age of father in years (mean ± SD)	41 ± 9.1	
Education of father		
Below university	173	45.1
University and above	211	54.9
Monthly income of the family (SR)		
1,000–3,000	29	7.6
3,001–5,000	33	8.6
5,001–7,000	57	14.8
7,001–9,000	80	20.8
>9,000	185	48.2
Number of children (mean ± SD)	2.7 ± 1.7	
Child with disability/chronic disease		
No	361	94.0
Yes	23	6.0
History of febrile convulsion		
No	342	89.1
Yes	42	10.9

Table 2 presents parents’ knowledge about children’s fever. The majority of interviewed parents responded correctly to the knowledge-related question regarding normal temperature, the definition of fever, and the definition of high fever (94.8%, 85.7%, and 83.3%, respectively). However, 56.8% knew the harmful degree of fever, and only 37% of parents perceived their knowledge about the management of childhood fever as adequate. The mean total knowledge score was 3.7 ± 1.1, with a median score of 4 (ranging from 0 to 5). Approximately 50% of the parents obtain their information about diagnosis and management of fever from doctors and 31.3% from the internet.

**Table 2:** Knowledge of parents about fever and their source of knowledge. SD: standard deviation; IQR: interquartile range

	Frequency N = 384	Percent
Knowing normal temperature		
Correct	364	94.8
Not correct	20	5.2
Defining fever		
Correct	329	85.7
Not correct	55	14.3
Defining high fever		
Correct	320	83.3
Not correct	64	16.7
Defining the harmful degree of fever		
Correct	218	56.8
Not correct	166	43.2
Perceived adequacy of knowledge to manage child’s fever at home		
Not adequate	242	63.0
Adequate	142	37.0
Total knowledge score, mean ± SD, median (IQR)	3.7 ± 1.1, 4 (3–4)	
Main source of information		
Doctor	190	49.5
Internet	120	31.3
TV	14	3.6
Written health materials	23	6.0
Relatives and friends	37	9.6

Table 3 showed that the majority of parents (77.1%) believed that fever will harm their children. About 45.8% of parents were very worried when their child had a fever and about 21.4% were extremely worried. Their main cause of worry was that fever may harm their children’s bodies, followed by the perception that the child with fever had low immunity (22.8%).

**Table 3:** Parents’ worry level and concerns about children’s fever. *Total less than 384 (25 responders had no worry).

Item	Frequency (N = 384)	Percent
Do you think fever is harmful to your child?		
No	88	22.9
Yes	296	77.1
Parent’s worry level		
Unworried	25	6.5
Little worried	101	26.3
Very worried	176	45.8
Extremely worried	82	21.4
The main cause of worry when the child has a fever*		
Fever indicates serious illness	65	18.2
Fever harms my child’s body	171	47.7
My child’s immunity may be low	80	22.8
Fever may cause seizures	43	12.3

Data concerning parental practice regarding home management of their febrile children showed wide variability (Table 4). Mostly 85.9% of parents measured their children’s temperature using a thermometer, and 55.2% of parents visited the hospital to measure their children’s temperature. They checked their child’s temperature at least every hour or more in about 18.5% of cases, more than 71.9% applied cold compression, 76.6% of parents gave their children non-prescribed fever medication, 58.1% encouraged their children to drink lots of fluids, 61.2% consulted their friends and families, and 51.6% of parents took their children to the doctor immediately irrespective of the degree of fever. Overall, 52.9% of parents gave their children more than one dose or one type before taking them to the doctor. Regarding the cause of consultation with a doctor, 90.1% were due to fever not responding to home management, 81.3% because there was no clear reason for the fever, 82.3% if children had other symptoms (runny nose, cough, diarrhea, and ear pain), 80.7% if the child was younger than three years, and 68.8% in case of high fever. The mean total practice was 9.3±2.2. More than half of the participants used paracetamol as an antipyretic while 24% used alternating doses of paracetamol and Ibuprofen. More than half of them had difficulties in giving the appropriate dose and frequency of administration of the antipyretic.

**Table 4:** Parents’ practice regarding the home management of childhood fever. SD: standard deviation; IQR: interquartile range

Practice domain	Frequency (N = 384)	Percent
Way of measurement of child’s temperature		
By hand	54	14.1
Using thermometer	330	85.9
Going to the hospital to measure temperature		
No	172	44.8
Yes	212	55.2
Frequency of temperature check		
<1 hour	313	81.5
≥1 hour	71	18.5
Application of cold compressors		
No	108	28.1
Yes	276	71.9
Use of antipyretics		
No	90	23.4
Yes	294	76.6
Increase in fluid intake		
No	161	41.9
Yes	223	58.1
Take advice from relatives or friends		
No	149	38.8
Yes	235	61.2
Take the child to the hospital irrespective of the degree of fever		
No	186	48.4
Yes	198	51.6
Consult doctors when the fever does not respond to home management		
No	38	9.9
Yes	346	90.1
Consult the doctor when there is no clear reason for the fever		
No	72	18.8
Yes	312	81.3
Give more than one dose or one type of antipyretic		
No	181	47.1
Yes	203	52.9
Consult the doctor when the fever is associated with other symptoms		
No	68	17.7
Yes	316	82.3
Consult the doctor when the child is younger than three years		
No	74	19.3
Yes	310	80.7
Consult the doctor when the child has a high fever		
No	120	31.3
Yes	264	68.8
Total practice score, mean ± SD, median (IQR)	9.3 ± 2.2, 10 (8-11)	
Type of antipyretic used		
Paracetamol	197	51.3
Ibuprofen	94	24.5
Both	93	24.2
Difficulties encountered by parents in administering antipyretics		
Difficulty in choosing the medicine (generic/brand)	152	39.6
Difficulty in deciding the correct dose	204	53.1
Difficulty in deciding how frequent the medicine should be given	196	51.0

**Figure 1:**
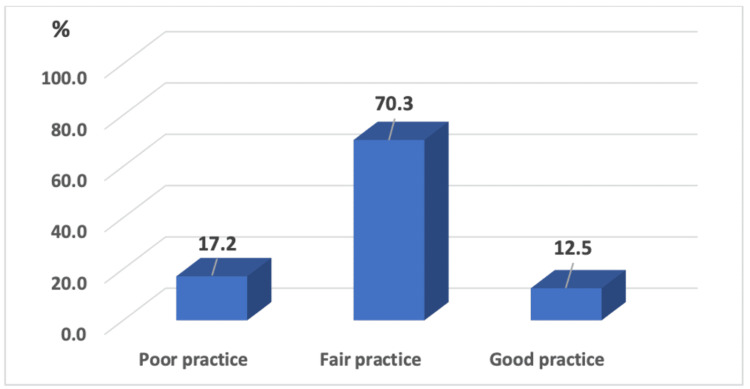
Distribution of participants according to the levels of adequacy in their practice regarding home management of fever in children.

Figure 1 shows that the majority (70%) reported fair practice and only 12% reported good practice.

Table 5 shows the factors affecting the knowledge and practice of parents regarding fever. The significant factors for knowledge of parents were baby care, education level of the mother, age of the mother and the father, and the main source of information. As a baby-care provider, the mother had significantly higher knowledge than the father and sister (p = 0.046). Both the age of the mother and the father had a significant weak correlation with knowledge score (Spearman correlation coefficient r_s_ = 0.113, p = 0.027 and r_s_ = 0.103, p = 0.044, respectively). Concerning the mother’s education level, mothers with a university or above education level had a significantly higher knowledge score than those with a below university-level education (p = 0.044). The main source of information significantly affected the knowledge score, and the pairwise comparison revealed that both parents who received information mainly from TV or relatives and friends had significantly lower average knowledge scores than those who received information from doctors, the internet, or written health materials.

**Table 5:** Factors associated with knowledge and practice of parents regarding childhood fever. *Significant at p ≤ 0.05; r_s_= Spearman correlation coefficient. SD: standard deviation; IQR: interquartile range

Characteristics	Knowledge score				Practice score		
	Mean (SD)	Median (IQR)	P-value		Mean (SD)	Median (IQR)	P-value
Gender							
Male	3.3 (0.9)	3 (4-3)	0.281		9.2 (2)	9 (8-10)	0.121
Female	3.4 (1.2)	4 (4-3)			9.4 (2.3)	10 (8-11)	
Baby care			0.046*				0.064
Mother	3.5 (1.1)	4 (4-3)			9.4 (2.3)	10 (8-11)	
Father	3.1 (1.1)	3 (4-3)			8.9 (1.2)	9 (8-11)	
Sister	3.2 (0.9)	3 (3-2.7)			9.3 (1.4)	9.5 (8-10)	
Mother’s age	r_s_ = 0.113, p = 0.027*			r_s_ = 0.106, p = 0.038*			
Mother’s education level							
Below university	3.2 (1.1)	3 (4-3)	0.044*		8.8 (2.5)	9 (8-11)	0.002*
University and above	3.5 (1.1)	4 (4-3)			9.7 (2.1)	10 (9-11)	
Father’s age	r_s_ = 0.103, p = 0.044*				r_s_ = 0.064, p = 0.214		
Father’s education level			0.360				0.580
Below university	3.4 (1.1)	4 (4-3)			9.3 (2.3)	10 (8-11)	
University and above	3.5 (1.1)	4 (4-3)			9.4 (2.3)	10 (8-11)	
Income of family (monthly) SR							
1,000–3,000	3.1 (1.2)	3 (4-2)	0.360		9.3 (2)	9 (9-10)	0.530
3,001–5,000	3.7 (1.3)	4 (4-3)			9.4 (2.7)	10 (8.5-11)	
5,001–7,000	3.4 (1.3)	4 (4-2)			9.7 (2.1)	10 (9-11)	
7,001–9,000	3.4 (1.2)	4 (4-3)			8.9 (2.5)	9 (8-11)	
>9,000	3.5 (1)	4 (4-3)			9.4 (2.2)	10 (8-11)	
Number of children	r_s _= -0.065, p = 0.206				r_s_ = -0.095, p = 0.064		
Children with disability or chronic disease							
No	3.4 (1.1)	4 (4-3)	0.521		9.3 (2.2)	10 (11-8)	0.584
Yes	3.4 (1.4)	4 (4-3)			8.8 (2.7)	10 (11-6)	
History of febrile convulsion							
No	3.4 (1.1)	4 (4-3)	0.216		9.4 (2.3)	10 (11-8)	0.666
Yes	3.5 (1.2)	4 (4-3)			9.2 (2.1)	10 (11-8)	
Main source of information			0.005*				0.005*
Doctor	3.6 (1.1)	4 (4-3)			9.6 (2)	10 (11-8)	
Internet	3.4 (1.01)	3.5 (4-3)			9.4 (2.3)	10 (11-9)	
TV	2.3 (1.3)	2 (3-1)			7.2 (3.1)	8 (9-6.5)	
Written health materials	3.3 (1.3)	4 (4-3)			9.3 (2.8)	10 (12-8)	
Relatives and friends	3.1 (1.2)	3 (4-2)			8.7 (2.5)	9 (11-8)	
Parent’s worry level							
Unworried	3.6 (0.9)	3 (3-4.5)	0.123		10.6 (2.1)	10 (9-12)	0.011*
Little worried	3.3 (1.1)	3 (3-4)			9.3 (2.3)	10 (8-11)	
Very worried	3.6 (1.1)	4 (3-4)			9.4 (2.04)	10 (8-11)	
Extremely worried	3.3 (1.1)	3.5 (3-4)			8.9 (2.6)	9 (8-11)	
Spearman correlation between knowledge and practice	r_s_ = 0.402, p = 0.000*						

Concerning the practice of parents, the significant factors were the mother’s age, mother’s education level, the source of information, and parent’s worry level. The age of the mother had a weak significant correlation and practice score (Spearman correlation coefficient r_s _= 0.106, p = 0.038). Regarding mother education level, mothers with a university education or above had significantly higher practice scores than those with education below university level (p = 0.002).

The main source of information significantly affected the practice score, and the pairwise comparison revealed that both parents who received information mainly from TV or relatives and friends had significantly lower average practice scores than those who received information from doctors, the internet, or written health materials. Regarding parent worry level, unworried parents had a significantly higher practice score than those who were little worried, very worried, and extremely worried (p = 0.011). There was a significant direct intermediate correlation between total knowledge score and total practice score (r_s_ = 0.402, p = 0.000).

Table 6 shows the results of the linear regression; the significant predictors of parent knowledge were the main source of information such as doctors (B = 1.386), the internet (B = 1.228), written health materials (B = 1.073), and mother’s university or above education (B = 0.386) in the order of increasing level of knowledge. Regarding the practice of parents, the most important significant predictor was the level of parental worry where it was negatively affecting the practice with all three levels of worry, namely, little, very, and extreme worry (B = -1.357, -1.706, and -2.059, respectively). The second most important predictor was the source of information as doctors and the internet were both positively affecting the practice score (B = 1.315 and 1.253, respectively). The third and fourth significant predictors were knowledge of participants regarding fever and university-level education of mothers and above where both positively affect the practice (B = 0.812 and 0.753, respectively).

**Table 6:** Significant predictors of parents’ knowledge and practice regarding the management of childhood fever Model 1: F = 6.3, P = 0.000, R^2^ = 0.363. Model 2: F = 15.5, P = 0.000, R^2 ^= 0.294.

Significant predictors	B	t	P-value
Model 1: knowledge			
Mother’s education (university/above)	0.306	2.469	0.014
Source of information			
Doctor	1.386	4.593	0.000
Internet	1.228	3.997	0.000
Written material	1.073	2.904	0.004
Model 2: practice			
Mother education (university/above)	0.753	3.488	0.001
Source of information			
Doctor	1.315	2.333	0.020
Internet	1.253	2.217	0.027
Level of worry			
Little worry	-1.357	-3.123	0.002
Very	-1.706	-4.066	0.000
Extreme worry	-2.059	-4.585	0.000
Total knowledge score	0.812	8.783	0.000

## Discussion

Despite fever being a common condition among children, several studies have documented that parents have a low level of knowledge and poor practice regarding the management of childhood fever [3,6,15]. Because there is no reliable tool to assess those practices, we conducted this study aiming to assess the gap in knowledge and practice regarding fever among parents and its determinants in the Taif region using a reliable tool with good internal consistency (α = 0.760). The study showed that the average total knowledge score of participants was 3.7 out of a maximum of 5 points, indicating good knowledge about fever, which can be explained by the high percentage of university education among participants, especially mothers (65%) who constituted the majority of participants (78%). In addition, this high knowledge can be explained by the fact that the main source of knowledge was doctors among our participants. Hence, our results support this explanation that the source of knowledge and mothers’ education are the significant determinants of total knowledge score according to the multiple linear regression analysis. Similarly, Al Arifi reported that 79.2% of parents had good knowledge about the correct definition of fever and 92% could define normal temperature [4]. On the contrary, other studies have reported that identifying the degree of fever is a common problem, with 46% and 63.1% and >70% of participants being unable to identify fevers [1,8,16]

However, our participants had a low knowledge level regarding defining the harmful degree of fever, with 43.2% of participants responding incorrectly to this question. This issue is critical and needs corrective action, such as health education sessions stressing this point.

Parents obtain information to manage fever from different sources. In our study, about half of the parents (49.5%) gained information from doctors, followed by the internet. These findings are in line with previous studies which found that 67.8% of Italian parents rely on doctors for information on fever and how to treat it [6]. According to a study by Wilson et al. from Australia, health expert staff provide 56.8% of information regarding fever to parents [17]. These findings shed light on the crucial role of the health sector in raising awareness among parents about fever and how to manage it. Despite the availability of brief patient education leaflets in most clinics, their role as a source of information for parents is limited; hence, it is better to replace these with video education during waiting time.

Fever is a physiologic mechanism with beneficial effects in fighting infections and is not associated with long-term neurologic complications. The majority (77%) of our participants had a misconception that fever is harmful. Furthermore, their level of worry was high which may be attributed to the misbelief that fever may harm the child’s body or decline their immunity; this was in line with a previously reported study [18]. However, these findings differed from those of other studies conducted in Saudi Arabia, Jorden, and Kuwait [4,19,20], in which the most worrying causes were fear from a side effect of fever such as brain damage, followed by seizures. This difference may be attributed to the lower level of the mothers’ education in those studies in comparison to our study.

The most striking finding from our survey is the great variability in answers of parents related to the management of a febrile child. Moreover, it is striking that only 12% of our participants had good practice in the management of fever in children despite their high level of knowledge; hence, knowledge was a significant determinant of the increase in practice score in the multiple regression analysis. This can be explained by the presence of other factors which had a higher magnitude of effect, such as worry level which contributed to declining practice score. Other studies have reported that parents’ fever phobia leads to poor management and unnecessary measures to manage the fever. The unnecessary measures that were adopted by our participants were going to the hospital to measure temperature (55.2%), very frequent temperature checks in less than one hour (81.5%), and taking a child to the hospital irrespective of the degree of fever (51.6%). These non-appropriate practices may also result from low perceived adequacy of knowledge among parents which leads to overload on the healthcare system. In addition, parents administered more than one dose or one type of antipyretic that can lead to an overdose problem.

A thermometer is required to determine the presence of fever in a child; the majority of parents in our study used a thermometer to detect their children’s fever while only 14% use hand touch to the child’s forehead to determine the presence of fever. The thermometer was rarely reported to be used at home to measure children’s temperature in different studies. In India, only 15% used the thermometer [21], and 25% used a thermometer in Iran [22]. Due to a lack of thermometers, children may have their fevers overestimated, resulting in unnecessary antipyretic medication.

Physical means are recommended evidence-based practices to help reduce fever. These measures include adequate hydration and the application of a cold sponge. In our study, 71.9% of participants reported applying cold compressions. Similar practices were reported in a local study by AlAteeq et al. with 84% of participants [1].

In our study, 76.6% of participants administered non-prescription fever medicine. More than half of them administered paracetamol as an antipyretic in the treatment of childhood fevers, consistent with the findings of other studies in other populations [23,24]. However, our participants faced some difficulties related to the doses and frequency of administration of the antipyretic. In contrast, two previous studies showed that the parents’ knowledge and management practices toward childhood fever were significantly positively correlated with their age and how many children they had. This was attributed to the experience of parents [3,19]. Appropriate measures promote better health during the fever phase and reduce the number of medical visits. This shows the need for training by the media, physicians, and healthcare centers. While in healthy children a temperature up to 39°C does not require treatment with antipyretic medications, fear of side effects and lack of sufficient knowledge lead to the treatment of fever even at lower temperatures [25,26]. The results of the linear regression in this study confirm that the primary source of information (doctors, the internet, and written health materials) and the mother’s education level (university or higher) are the most relevant factors predicting knowledge level, as well as the level of parental worry, negatively affecting proper management practices toward childhood fever. Higher mother education and the information gained from doctors and the internet positively affect the practice. In addition, parental awareness reduces misunderstanding and ensures good practice in the management of fever.

Strength and limitations

The recruited parents were not under the pressure of an unwell child. Further, the use of a reliable questionnaire is a strength of this study, as well as the recruitment of a representative sufficient sample. However, cross-sectional studies have some limitations. Specifically, the collected data were observational and there may be a recall bias.

## Conclusions

Even though the majority of parents had good knowledge regarding fever among children, there is a need to correct the misconception that fever is harmful. Only 12% of surveyed parents had good practice regarding fever management. There is a significant percentage of parents who use non-prescribed antipyretic medication for their feverish children even for low-grade fevers. Parents experienced a high level of worry about the perceived potential harms of fever which lead to inappropriate practices and unnecessary visits to seek medical advice. This mandates the implementation of health education campaigns for the community on the management of fevers and recognizing the warning signs in a feverish child.

The parental practices were significantly related to a high level of mother’s education, main sources of information as doctors and the internet, as well as their worry level. The findings highlight the necessity of clinicians to communicate with parents in a straightforward and informed manner to alleviate their worry which will reduce their burden of care and overuse of health services during febrile episodes, as well as reduce unnecessary over-the-counter medications. Future studies for interventions to increase parental practice are needed.

## Appendices

The Questionnaire

Section 1: Demographic data

Section 2: knowledge

Section 3: Practice

Section 1: Demographic data

·       Age

·       Gender

·       Job

·       Father’s educational level

·       Mother’s educational level

·       Area/Neighborhood

·       Number of children

·       Income level of the family (monthly) SR:

•       1,000-3,000

•       3,001-5,000

•       5,001-7,000

•       7,001-9,000

•       9,000

•       Do you have any child with a disability or chronic disease? Yes or No

•       Was there any history of febrile convulsion in one of your children or any family member? Yes or No

Section 2: Knowledge and beliefs

1.     Please mark with an X on the line the temperature you would consider to be NORMAL for your child.

I_____ı_____I_____ı_____I______ı_____I_____ı_____I_____ı_____I_____ı_____I_____ı_____I_____ı

35.0°C               36.0°C              37.0°C               38.0°C              39.0°C              40.0°C              41.0°C              42.0°C

2.     Please mark with an X on the line the temperature you would consider to be fever for your child.

I_____ı_____I_____ı_____I______ı_____I_____ı_____I_____ı_____I_____ı_____I_____ı_____I_____ı

35.0°C               36.0°C              37.0°C               38.0°C              39.0°C              40.0°C              41.0°C              42.0°C

3.     Please mark with an X on the line the temperature you would consider to be high fever for your child.

I_____ı_____I_____ı_____I______ı_____I_____ı_____I_____ı_____I_____ı_____I_____ı_____I_____ı

35.0°C               36.0°C              37.0°C               38.0°C              39.0°C              40.0°C              41.0°C              42.0°C

4.     Do you think that fever is harmful to your child? (if No, go to question 8) yes or no.

5.     At what level do you think fever is harmful to your child?

I_____ı_____I_____ı_____I______ı_____I_____ı_____I_____ı_____I_____ı_____I_____ı_____I_____ı

35.0°C               36.0°C              37.0°C                38.0°C             39.0°C              40.0°C             41.0°C              42.0°C

6.  How much do you think fever is harmful to your child?

Very harmful

Harmful

Little harmful

7. If you think fever is harmful to your child, what is the harm you are concerned with? (List in order)

Febrile convulsion

Hearing loss

Dehydration

Brain damage

Loss of consciousness

Other organ damage (liver, kidney, etc.)

Others:

8. What is the primary source of your information for the diagnosis and management of fever at home?

Family doctor

TV

Internet

Children doctor

Written health materials

Relatives and friends 

Others:

9. Do you think you have enough information for diagnosing and managing your child’s fever at home?

Yes

No

Not sure

10. What are you most worried about when your child has a fever? (List in order)

Fever may indicate a serious illness

My child’s immunity may be low

Fever may harm my child’s body

Fever may cause seizures

Others:

Section 3: Practice

11. When you think your child has a fever, you (mostly, sometimes, never)

Measure it manually (by hand)

Use oral thermometer

Use axillary thermometer

Use rectal thermometer

Take him to the health center/hospital/ER

12. When your child has a fever, you (mostly, sometimes, never)

Apply cold compressions

Give him/her fever medicine

Give him/her plenty of fluids

Consult a relative or friend

Take him/her to the doctor right away

13. When giving your kid medication to reduce fever at home, you usually (mostly, sometimes, never)

Give medication only for high fever

Give medication for fever, regardless of its level

Give more than one dose before taking him to the doctor

Give more than one medicine at the same time (different types)

14. What makes you decide to take your child to the doctor/health center/emergency department for fever? (yes or No)

If he/she has a fever, regardless of its level

If he/she has a high fever

If not responding to fever medicine

If no improvement within 24 hours

If there is no clear reason for fever (cold/diarrhea/teething, etc.)

If he/she has other symptoms (runny nose/cough/diarrhea/ear pain/drowsiness, etc.)

If he/she is younger than 3 years

15. When giving medicine to reduce fever in your kid, what medicine do you use? (mostly, sometimes, never)

Fevadol (syrup/suppository)

Tylenol (syrup/suppository)

Tempra (syrup/suppository)

Adol (syrup/suppository)

Brufen syrup

Others:

16. Do you have difficulty in giving your child medicine for fever as an over-the-counter medicine? (yes or no)

17. If you have difficulty in giving your kid medicine for fever at home, what is it? (list in order) (mostly, sometimes, never)

Choosing the medicine (generic/brand)

Deciding the correct dose

Deciding how frequent the medicine should be given

Others:
